# Ubiquitin-conjugating enzyme UBE2J1 negatively modulates interferon pathway and promotes RNA virus infection

**DOI:** 10.1186/s12985-018-1040-5

**Published:** 2018-08-29

**Authors:** Tingting Feng, Lei Deng, Xiaochuan Lu, Wen Pan, Qihan Wu, Jianfeng Dai

**Affiliations:** 1 Institute of Biology and Medical Sciences, Jiangsu Key Laboratory of Infection and Immunity, Soochow University, 199 Ren-ai Road, Suzhou, 215123 People’s Republic of China; 2 Key Laboratory of Reproduction Regulation of NPFPC, SIPPR, IRD, Fudan University, Shanghai Institute of Planned Parenthood Research, Shanghai, 200032 People’s Republic of China

**Keywords:** UBE2J1, Dengue virus, Interferons, IRF3, K48 ubiquitination

## Abstract

**Background:**

Viral infection activates innate immune pathways and interferons (IFNs) play a pivotal role in the outcome of a viral infection. Ubiquitin modifications of host and viral proteins significantly influence the progress of virus infection. Ubiquitin-conjugating enzyme E2s (UBE2) have the capacity to determine ubiquitin chain topology and emerge as key mediators of chain assembly.

**Methods:**

In this study, we screened the functions of 34 E2 genes using an RNAi library during Dengue virus (DENV) infection. RNAi and gene overexpression approaches were used to study the gene function in viral infection and interferon signaling.

**Results:**

We found that silencing UBE2J1 significantly impaired DENV infection, while overexpression of UBE2J1 enhanced DENV infection. Further studies suggested that type I IFN expression was significantly increased in UBE2J1 silenced cells and decreased in UBE2J1 overexpressed cells. Reporter assay suggested that overexpression of UBE2J1 dramatically suppressed RIG-I directed IFNβ promoter activation. Finally, we have confirmed that UBE2J1 can facilitate the ubiquitination and degradation of transcription factor IFN regulatory factor 3 (IRF3).

**Conclusion:**

These results suggest that UBE2 family member UBE2J1 can negatively regulate type I IFN expression, thereby promote RNA virus infection.

**Electronic supplementary material:**

The online version of this article (10.1186/s12985-018-1040-5) contains supplementary material, which is available to authorized users.

## Background

Dengue virus (DENV), transmitted by *Aedes aegypti* and *Aedes albopicuts*, causes an emerging tropical disease that arouses increasing public concern in recent years [1, 2]. Up to 1.5 million infected individuals present with clinical symptoms and ~ 500,000 infections progress to the life-threatening dengue hemorrhagic fever and dengue shock syndrome. No specific treatment for dengue infection is available at the moment.

Viral infection activates innate sensing pathways. Interferons (IFNs) play a pivotal role in the outcome of a viral infection, and regulate both innate and adaptive antiviral responses [3]. IFN-α/β regulates the synthesis of antiviral proteins and immunoregulatory factors through the JAK/STAT signaling pathway [4, 5]. A lot of host and viral proteins can regulate the type I interferon pathway thereby significantly influence the progress of virus infection.

Ubiquitin modifications of proteins within the signaling cascades induce type I interferon expression, and in contrast, some viruses are found to utilize the ubiquitin system to suppress IFNs [6, 7]. Among them, classical K48-linked polyubiquitin chains mediate the target proteins for proteasomal degradation, while K63-linked polyubiquitination usually stabilize the target proteins and further activate their function. K48-linked polyubiquitin chains ligated the CARD domain of RIG-I and MDA5, leading to proteasome-mediated degradation of both receptors and repressing IFN-I signaling [8]. In contrast, K63-linked ubiquitination positively regulates signal transduction events and does not induce protein degradation [9]. In addition to RIG-I, TRAF3/IKKε/IRF3/7 in the type I interferon signaling pathway and NEMO/IKKα/RIP1 in the NF-κB signaling pathway have also been reported to be regulated by different types of ubiquitination [10, 11].

Ubiquitination is catalyzed by a series of ubiquitin related enzymes, which are the ubiquitin-activating enzyme (E1), ubiquitin-conjugating enzyme (E2), ubiquitin protein ligase (E3) and deubiquitinating enzymes (Dubs). Generally, the E3 determined the substrate specificity, while the E2s have the capacity to determine ubiquitin chain topology and emerged as key mediators of chain assembly [9].

E2 enzymes direct the ubiquitination process to distinct subsets of ubiquitin lysines. Different E2 enzymes influence distinct types of ubiquitin linkages. For example, the E2 enzyme UBE2N (also known as Ubc13) heterodimerizes with UBE2V1, and mediates K63-linked ubiquitination [12, 13]. UBE2L3 promotes K48-ubiquitylation of pro-IL-1β and dampens mature-IL-1β production [14]. Cdc34 had a strong preference for K48, with lower selectivity towards K11 and K63 [15]. UBE2F, combined with SAG/CUL5 complex, activates CRL5 (Cullin-RING-ligase-5) and ubiquitylates NOXA via a novel K11 linkage which leads to proteasomal degradation [16]. In addition to ubiquitination, some other E2s could mediate ubiquitin-like protein modification. Ubc9 (UBE2I) is the central enzyme in SUMO conjugation system. It forms a SUMO~Ubc9 thioester bond then transfer SUMO to a target protein substrate in the presence of an E3 [17]. In a mechanism similar to ubiquitination, ISG15 is conjugated to targets via the sequential co-operation of E1, E2 and E3 enzymes, in which the specific E2 was UBE2L6 [18].

There are around 40 ubiquitin E2 family genes in human genome, and a lot of them are associated with pathologies and diseases [9]. Since E2s plays important roles in determining the topology of ubiquitin chains and the fates of the proteins modified, we screened 34 UBE2 genes using an RNAi library and investigated the potential role of individual E2 during DENV infection. Here, we reported UBE2J1 as a negative regulator of IFN pathway by mediating IRF3 ubiquitination and degradation.

## Methods

### Virus, cell culture and infection

DENV-2 virus (DENV New Guinea C stain) and Zika virus (ZIKV, MR766 strain) were propagated in mosquito C6/36 cells (ATCC® CRL-1660). Influenza A virus (H1N1-A/PR/8/34) and Sendai virus (SeV) was propagated in 10 days old embryonated chicken eggs (Bejing Laboratory Animal Research Center, Beijing, China), and titrated by hemagglutination assay using chicken red blood cells (BeNa Culture Collection, Bejing, China).

Human epithelial cell line HEK293T cells, A549 cells, and human Peripheral Blood Mononuclear Cells (PBMCs) were cultured in DMEM or RPMI 1640 medium supplemented with fetal bovine serum (10%) and penicillin/streptomycin (1%). HEK293T cells were infected with DENV, ZIKV, SeV or H1N1 at a multiplicity of infection (MOI) of 1, unless otherwise stated.

### Plasmid constructs

Recombinant plasmid for UBE2J1 expression was constructed using standard protocols by inserting the UBE2J1 open reading frame into the pcDNA3.1 vector. Expression plasmids for the active caspase recruitment domain (CARD) containing form of RIG-I (RIG-I-N), MAVS, TBK1, IKKε and the constitutively active IRF3 (IRF3-5D), Luciferase reporter plasmids IFNβ-Luc and IRF3-Luc were kindly provided by Dr. Rongtuan Lin, McGill University, Canada [19].

### RNAi and transfections

HEK293T cells (~ 5 × 10^5^) were transfected with 500 ng of plasmid DNA or si/shRNA using Lipofectamine 2000 (Invitrogen, USA) according to the manual of the manufacturer. 24 h post transfection, the cells were infected with DENV at an MOI of 1 for another 48 h (unless otherwise stated). The UBE2s siRNA library for 34 genes was purchased from RiboBio Co., China. The shRNA sequences for human *UBE2J1* gene were 5’-GTGAAGAGTCCGGCTGTTA-3′, and 5’-TAACAGCCGGACTCTTCAC-3′. Quantitative reverse transcription polymerase chain reaction (qRT-PCR) was used to confirm the RNAi efficiency of specific gene.

### RNA isolation and qRT-PCR

Total RNA were extracted using the total RNA kit (OMEGA, USA) and reverse-transcribed using the PrimeScriptTM Master Mix kit (TaKaRa, Japan). The intracellular viral loads, in terms of transcript levels of the specific viral genes, were quantified through qRT-PCR and normalized to *β-actin* gene. (Oligo-primer sequences for qRT-PCR of this study were shown in “Additional file 1”).

### TCID_50_ assay and viral growth kinetics

The cell-free supernatants were collected and the titers of DENV and ZIKV were determined with a median tissue culture infective dose (TCID_50_) assay according to standard protocols on Vero cells [20, 21]. Briefly, samples were serially diluted and inoculated into Vero cells in 96-well plates. After 5-day incubation, cells were examined for cytopathic effects (CPE) under a light microscope. The virus titer (TCID_50_/ml) was calculated using the Reed-Muench method. The titers of H1N1 and SeV were measured by hemagglutination assay using the chicken red blood cells [22].

### Luciferase reporter assays

Luciferase reporter assays were performed as described previously [23]. Briefly, 70% confluent HEK293T cells were transfected with 10 ng of pRL-TK reporter (*Renilla* luciferase, internal control), 100 ng of IFNβ luciferase reporter (firefly luciferase, experimental reporter), 50 ng of IFNβ activators (RIG-I-N, MAVS, TRAF3, TBK1, IKKε, or IRF3-5D), as well as either 100 ng of recombinant over-expressing or shRNAs plasmids (Vector, UBE2J1, scramble shRNAs or UBE2J1 shRNA). For measuring the activation of transcription factor IRF3, HEK293T cells were transfected with IRF3 specific luciferase reporter pIRF3-Luc (pRD (III–I)-Luc) plasmid instead of the IFNβ luciferase reporter. At 24 h post-transfection, luciferase activity was measured using a Promega Dual Glow kit according to the instructions of the manufacturer.

### Western blot

HEK293T cells (~ 5 × 10^6^) were transfected with UBE2J1 overexpressing or silencing plasmids and then infected with DENV. At indicated time points post-infection, cell lysates were subjected to SDS-PAGE and transferred onto a PVDF membrane for western blotting. The following antibodies were used for western blotting: anti-human GAPDH polyclone antibody (Proteintech, USA), His-tag polyclone antibody (GenScript, USA), HA-probe mouse monoclonal antibody (Santa Cruz, USA), anti-Flag-tag (Sigma-Aldrich, USA), RIG-I pathway antibody sampler kit (Cell Signaling Technology, USA), IRF3 polyclonal antibody (Biolegend, USA), HRP-conjugated donkey anti-rabbit IgG and rabbit anti mouse IgG mAb (Biolegend, USA). The signals were detected using an Enhanced Chemiluminescent (ECL) kit according to the manufacturer’s instructions (Merck Millipore Ltd., USA).

### Enzyme linked immunosorbent assay (ELISA)

HEK293T Cells were transfected with UBE2J1 overexpressing or silencing plasmids and then infected with DENV. At indicated time points post-infection, IFNβ protein in the cell supernatant were determined by ELISA kit (PBL Assay Science, USA) according to the manufacturer’s instructions. .

### Ubiquitination and Coimmunoprecipitation assays

HEK293T cells (~ 5 × 10^6^) were transfected with Flag-IRF3, His-UBE2J1 (or vector), together with HA-ubiquitin, HA-K48 ubiquitin or HA-K63 ubiquitin plasmids (Addgene, USA), respectively. 48 h later, cells were treated with proteasome inhibitor MG132 (20 μM, Sigma-Aldrich, USA) for another 6 h before harvest.

Immunoprecipitation were performed with anti-Flag® M2 Affinity Gel (Sigma-Aldrich, USA) according to the manufacturer’s instructions. Briefly, 500 μl of cell lysates were incubated with 100 μl of the anti-Flag M2 Affinity Gel overnight at 4 °C. The beads were washed four times with 1 ml of lysis buffer containing 500 mM NaCl. The immunoprecipitated Flag-IRF3 proteins were then eluted and subjected to western blots. The co-immunoprecipitated UBE2J1 and ubiquitination signal on Flag-IRF3 was detected using anti-His and anti-HA antibodies, respectively.

### Statistical analysis

Statistical significances were calculated with an unpaired two tailed Student’s *t*-test using Prism 6 software (GraphPad).

## Results

### Roles of UBE2 family during DENV infection

**Fig. 1:**
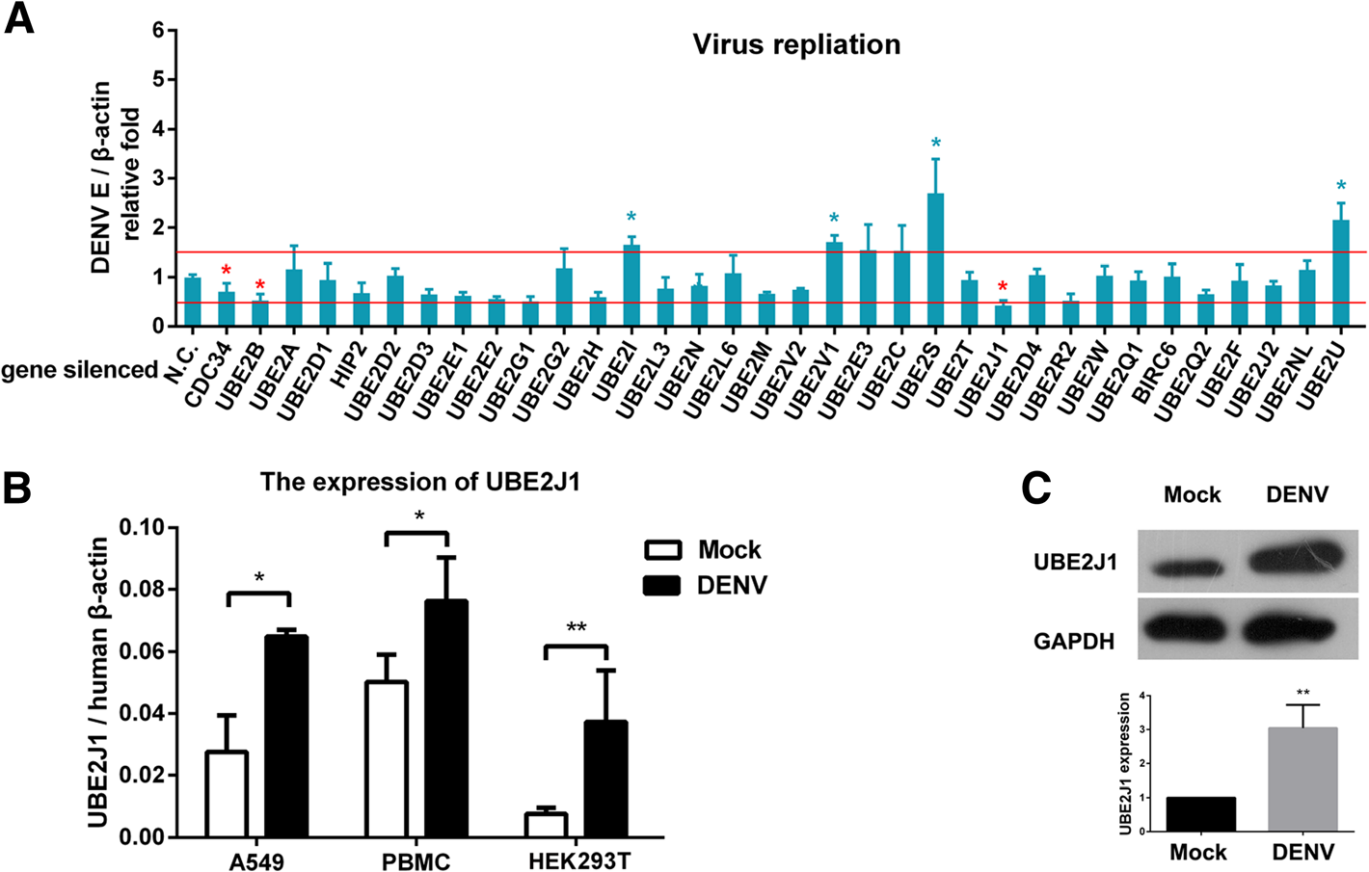
UBE2J1 is involved in DENV infection. **a** RNAi screening of UBE2 family during DENV infection: HEK293T cells were transfected with siRNAs for each specific gene of UBE2 family, and infected with DENV for 48 h at an MOI = 1. The viral loads were analyzed by measuring the DENV E gene copy using qRT-PCR and normalized to a human *β-actin* gene. The relative replication level of DENV in nonsense siRNA (N.C.) transfected cells was set to 1. The red lines were set to mark the significant suppression (fold change < 0.5) or stimulation (fold change > 1.5) of DENV RNA replication. Results were expressed as the mean + SD. Each siRNA treatment was compared to N.C. group individually: * *p* < 0.05 and ** *p* < 0.01 (*t*-test). **b** The qRT-PCR analysis of *UBE2J1* mRNA expression in A549, PBMCs, and HEK293T before and after DENV infection. Results were expressed as the mean + SD. * *p* < 0.05 and ** *p* < 0.01 (*t*-test). (C) Immunoblots analysis of UBE2J1 expression in HEK293T cells before and after DENV infection. The relative grey density of immunoblots bands (UBE2J1/β-actin) was analyzed by ImageJ software. Representative results from at least 3 independent experiments

RNAi screening of 34 human UBE2 genes were performed on HEK293T cells to investigate which UBE2s are involved in DENV replication. The DENV viral replications were analyzed by measuring the copies of viral mRNA transcripts (Envelope (E) gene) though qRT-PCR in each UBE2 siRNA treated HEK293T cells at 48 h post infection. Among them, DENV replication were significantly decreased in CDC34, UBE2B and UBE2J1 siRNA-treated cells, while increased in UBE2S, UBE2U, UBE2V1 and UBE2I siRNA-treated cells (*t*-test, *p* < 0.05 Vs NC) (Fig. 1a). Since UBE2J1 silencing caused a most dramatic decrease on DENV replication, we focused on the role of UBE2J1 on DENV infection in this study.

### UBE2J1 is upregulated during DENV infection

By using qRT-PCR, we noted an increased *UBE2J1* mRNA expression in DENV stimulated human A549, PBMCs, and HEK293T cells (Fig. 1b). Consistently, the protein level of UBE2J1 was also induced in HEK293T cells after DENV infection (Fig. 1c). These data suggest that UBE2J1 may involve in DENV infection of these cells.

### UBE2J1 promotes RNA virus replication

**Fig. 2:**
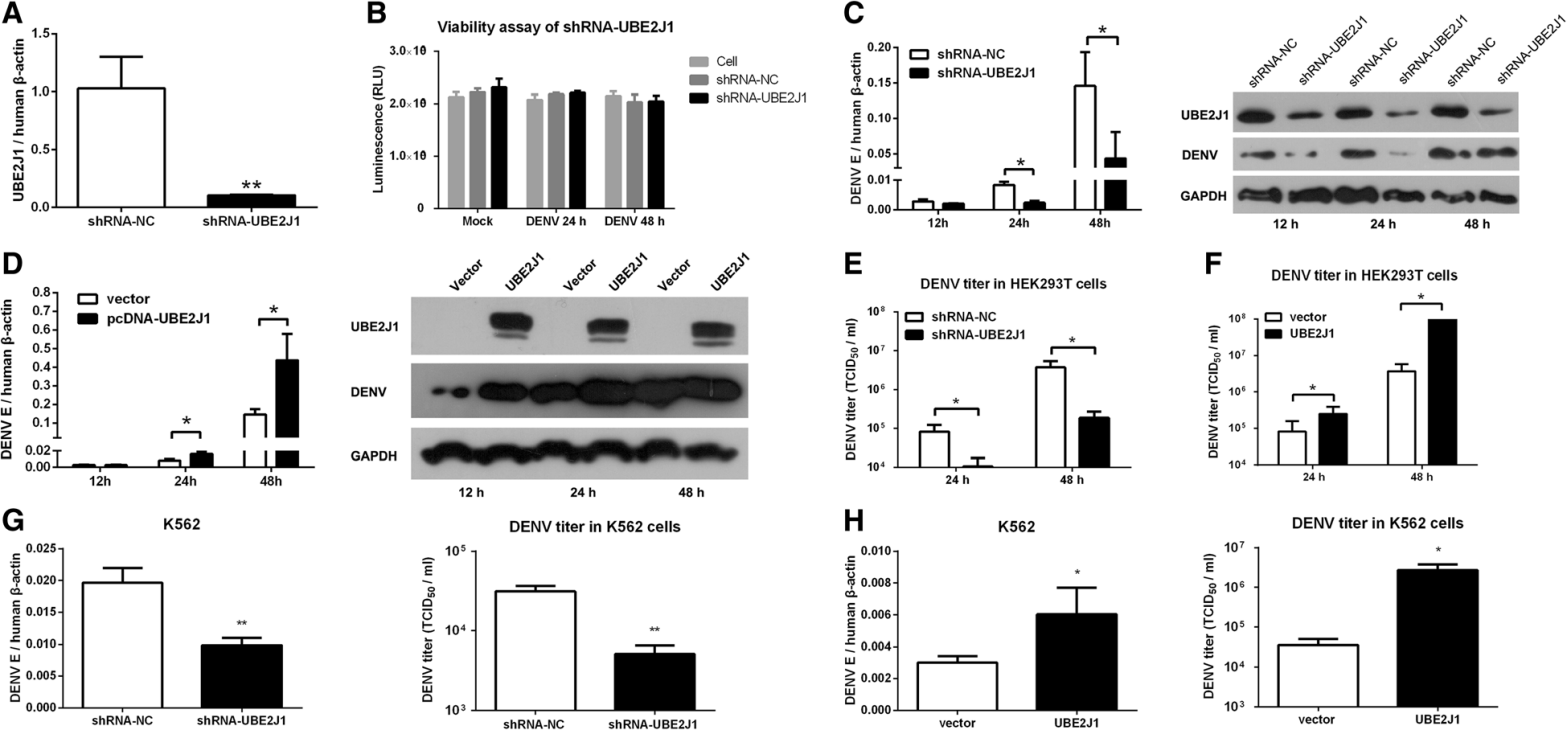
UBE2J1 promotes DENV infection. **a** RNAi efficiency of UBE2J1 shRNA in HEK293T cells. **b** Silencing UBE2J1 by RNAi and infecting cell with DENV for 24 h or 48 h (MOI of 1) showed no cytotoxic effect to HEK293T cells (Cell Viability assay, Promega). **c** DENV viral loads in UBE2J1 shRNA (or scramble shRNA, N.C.) treated HEK293T cells at 12 h, 24 h, and 48 h post DENV infection (MOI of 1). The viral burdens were analyzed by measuring the virus E gene expression using qRT-PCR and western blot, and normalized to human *β-actin* or GAPDH, respectively. **d** qRT-PCR and western blot analysis of DENV E gene expression in HEK293T cells with or without UBE2J1 overexpression at 12 h, 24 h, and 48 h post DENV infection. **e** and **f** Viral titers in supernatants of DENV infected HEK293T cells determined by TCID_50_ assay on Vero cells. **g**-**h** qRT-PCR analysis of viral mRNAs and TCID_50_ assay of viral titers in K562 cells transfected with UBE2J1 shRNA or UBE2J1 overexpression plasmids post DENV infection. Results were expressed as the mean + SD. * *p* < 0.05 and ** *p* < 0.01 (*t*-test). Representative results from at least 3 independent experiments

To confirm the role of endogenous UBE2J1 in DENV infection, UBE2J1 expression was silenced using RNAi approach. shRNA specifically targeting *UBE2J1* significantly suppressed *UBE2J1* mRNA expression compared with cells receiving scramble shRNA (N.C.) (Fig. 2a). Cell viability was not influenced during RNAi and DENV infection process (Fig. 2b) (DENV does not cause cytopathic effect in HEK293T cells). The viral replication efficiency, in terms of the mRNA copies the DENV E gene, decreased by 1.3-fold, 3.2-fold and 3.4-fold (*p* < 0.05) in UBE2J1-silenced cells compared with control cells at 12, 24 and 48 h post DENV infection, respectively (Fig. 2c). The protein levels of DENV E gene were also decreased in UBE2J1 silenced cells compared with controls at indicated time points post-infection (Fig. 2c). Conversely, we noted a significant increase in viral loads (~ 3 fold) in *UBE2J1* overexpressed HEK293T cells (Fig. 2d). Meanwhile, the titers of DENV in cell supernatants, as determined by TCID_50_ assay, were significantly decreased in UBE2J1 silenced cells (Fig. 2e) and increased in UBE2J1 overexpressed cells (Fig. 2f). Similar results were obtained in DENV infected K562 cells (Fig. 2g and h), which suggesting that UBE2J1 also involved in DENV infection of immune cells. Overall, these data suggest that UBE2J1 could promote DENV replication.

**Fig. 3:**
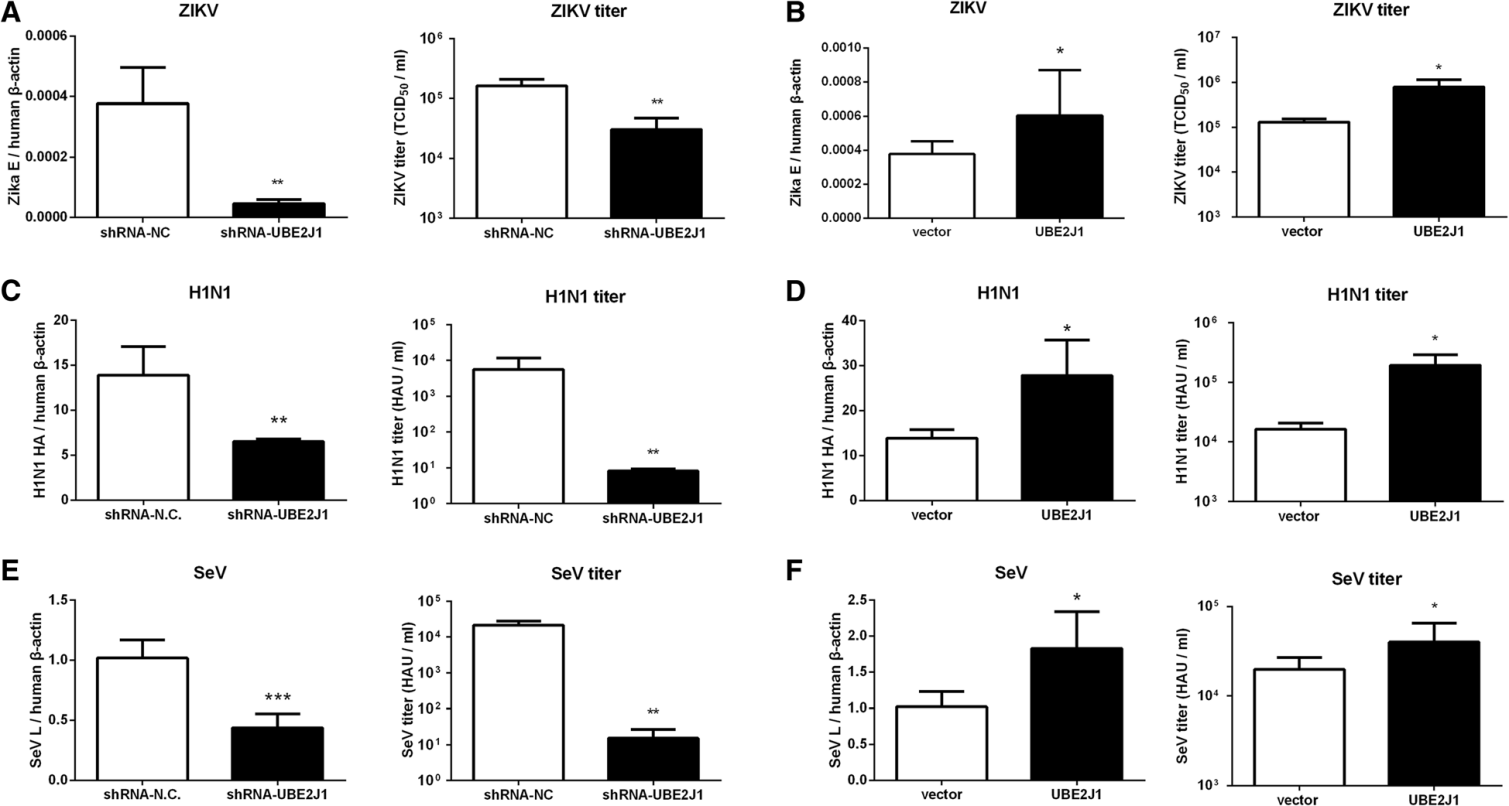
UBE2J1 promotes ZIKV, H1N1 and SeV infection. **a**-**f** viral mRNA copies and viral titers of ZIKV (**a** and **b**), H1N1 (**c** and **d**) and SeV in (**e** and **f**) in UBE2J1 silenced (**a**, **c**, and **e**) or overexpressed (**b**, **d**, and **f**) HEK293T cells compared with controls. HEK293T cells were transfected with UBE2J1 shRNA or UBE2J1 overexpression plasmids for 24 h, and then infected with ZIKV (MOI of 1 for 24 h), H1N1 (MOI of 1 for 24 h) or SeV (MOI of 1 for 16 h), respectively. The intracellular viral loads, in terms of transcript levels of the specific viral genes (Additional file 1: Table S1), were quantified through qRT-PCR and normalized to *β-actin* gene. The viral titers were determined by TCID_50_ assay (for ZIKV) or hemagglutination assay (for H1N1 and SeV, HA units (HAU) /ml). Results were expressed as the mean + SD. * *p* < 0.05 and ** *p* < 0.01 (*t*-test). Representative results from at least 3 independent experiments

Besides of this, UBE2J1 was found to promote replication of other viruses, including Zika virus (ZIKV), Influenza virus (H1N1) and Sendai virus (SeV) (Fig. 3). Viral mRNA copies and virus titers of ZIKV (Fig. 3a and b), H1N1 (Fig. 3c and d) and SeV (Fig. 3e and f) were significantly lower in UBE2J1 silenced cells while higher in UBE2J1 overexpressed cells, when comparing with controls. Interestingly, all these tested viruses are single-stranded RNA virus.

### UBE2J1 negatively regulates virus-induced IFNβ production

**Fig. 4:**
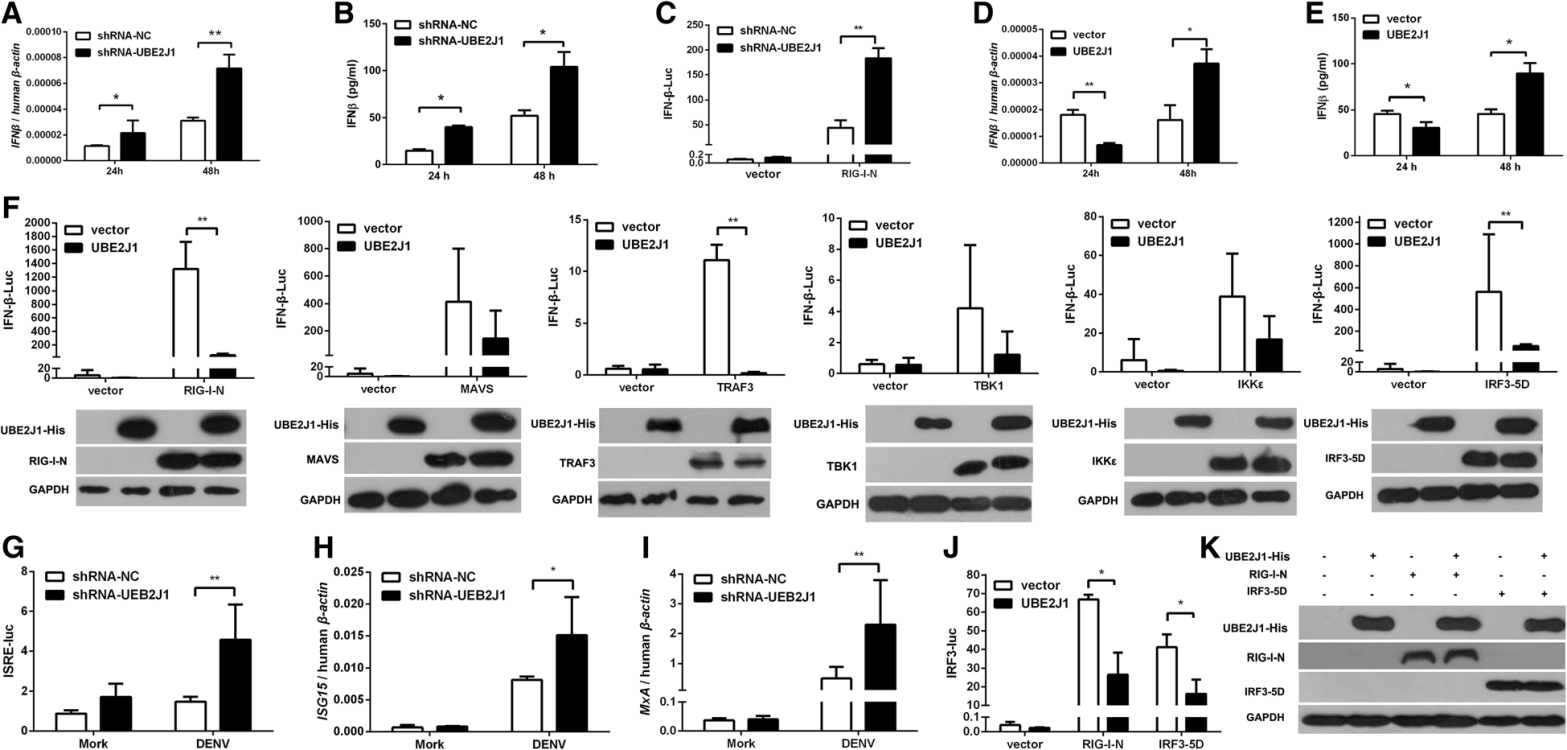
UBE2J1 negatively regulates IRF3-mediated IFNβ signaling. **a** *IFNβ* mRNA expression in scramble shRNA (N.C.) or UBE2J1 shRNA transfected HEK293T cells after DENV infection. **b** Protein levels of IFNβ in scramble shRNA (N.C.) or UBE2J1 shRNA transfected HEK293T cells after DENV infection. **c** HEK293T cells were co-transfected with the IFNβ luciferase reporter, RIG-I-N (or vector), pRL-TK and UBE2J1 (or N.C.) shRNA plasmids. At 24 h after transfection, luciferase reporter assay was used to analysis of IFNβ promoter activity. **d** HEK293T cells were transfected with vector or UBE2J1 overexpression plasmids and infected with DENV. At 24 h or 48 h post infection, *IFNβ* mRNA expression was analyzed by qRT-PCR. **e** DENV induced IFNβ protein in UBE2J1 overexpressing or control HEK293T cells as quantified using ELISA. **f** Effect of overexpression of UBE2J1 on IFNβ reporter activation directed by RIG-I-N, MAVS, TRAF3, TBK1, IKKε, or IRF3-5D. Western blots were performed to confirm the overexpression of indicated proteins. **g**-**i** UBE2J1 suppressed DENV induced ISRE-promoter activity and ISG mRNA production. HEK293T cells were transfected with UBE2J1 (or N.C.) shRNA and then treated with DENV (or Mock) infection. At 24 h post infection, ISRE promoter activities were analyzed by luciferase assay (**g**) and *ISG15* (**h**) and *MxA* (**i**) mRNA expression were measured by qRT-PCR. **j** Overexpression of UBE2J1 downregulated RIG-I-N, or IRF3-5D induced IRF3 promoter (IRF3-Luc) activity. **k** Western blots were used to confirm the overexpression of indicated proteins shown in data J. Results were expressed as the mean + SD. *p < 0.05 and ** *p* < 0.01 (*t*-test). Representative results from at least 3 independent experiments

Ubiquitination is reported as regulators of antiviral innate responses including the interferon signaling pathway. The aforementioned results clearly demonstrate a pro-vial role of UBE2J1 for several RNA viruses. We next evaluated whether UBE2J1 influence the innate immune responses to viral infection. The expression of *IFNβ* mRNA and protein were significantly increased at 24 h and 48 h post DENV infection in UBE2J1 silenced cells (Fig. 4a and b). Consistently, UBE2J1 silencing also enhanced the RIG-I directed IFNβ promoter-driven luciferase (IFNβ-Luc) expression (Fig. 4c). Conversely, we noted a decreased *IFNβ* expression at 24 h in UBE2J1-overexpressed HEK293T cells post DENV infection (Fig. 4d and e). However, the IFNβ was increased on 48 h after DENV infection with UBE2J1 overexpression (Fig. 4d and e). We hypothesized that this may be the secondary effect of the enhanced viral replication at late time point in those cells. These results suggest that UBE2J1 negatively regulates type one interferon signaling processes and suppresses early IFN production during virus infection.

RIG-I, MAVS, TBK1, IKKε, and IRF3 participate in IFN production during RNA virus infection. Therefore, we investigated the action point of UBE2J1 by dissecting its suppressive effects on these transducer proteins. UBE2J1-overexpressing plasmids, together with one of the IFN pathway activators, RIG-I-N, MAVS, TRAF3, TBK1, IKKε or IRF3-5D, were co-transfected into HEK293T cells. The expression these individual constructs of IFN pathway molecules activates IFNβ-Luc reporter activity. UBE2J1 was found to significantly inhibit IFNβ-Luc activity induced by RIG-I-N, TRAF3 and IRF3-5D (Fig. 4f).

Accordingly, the ISRE promoter activations were also induced when silencing UBE2J1 (Fig. 4g). Meanwhile, the mRNA levels of typical interferon stimulated genes, *ISG15* and *MxA,* were up-regulated and in consistent with the ISRE promoter activity (Fig. 4h and i).

To further validate whether UBE2J1 directly inhibits IRF3 activation, IRF3-specific luciferase activity was induced by coexpression of RIG-I-N or IRF3-5D. The IRF3 promoter activation (measured by an IRF3 specific reporter pIRF3-Luc (pRD (III–I)-Luc) [19]) was decreased in UBE2J1 overexpressed cells compared with controls (Fig. 4j and k), which implied that UBE2J1 could inhibit IRF3 transcriptional activity directly.

### UBE2J1 inhibits IFN production by ubiquitination of IRF3

**Fig. 5:**
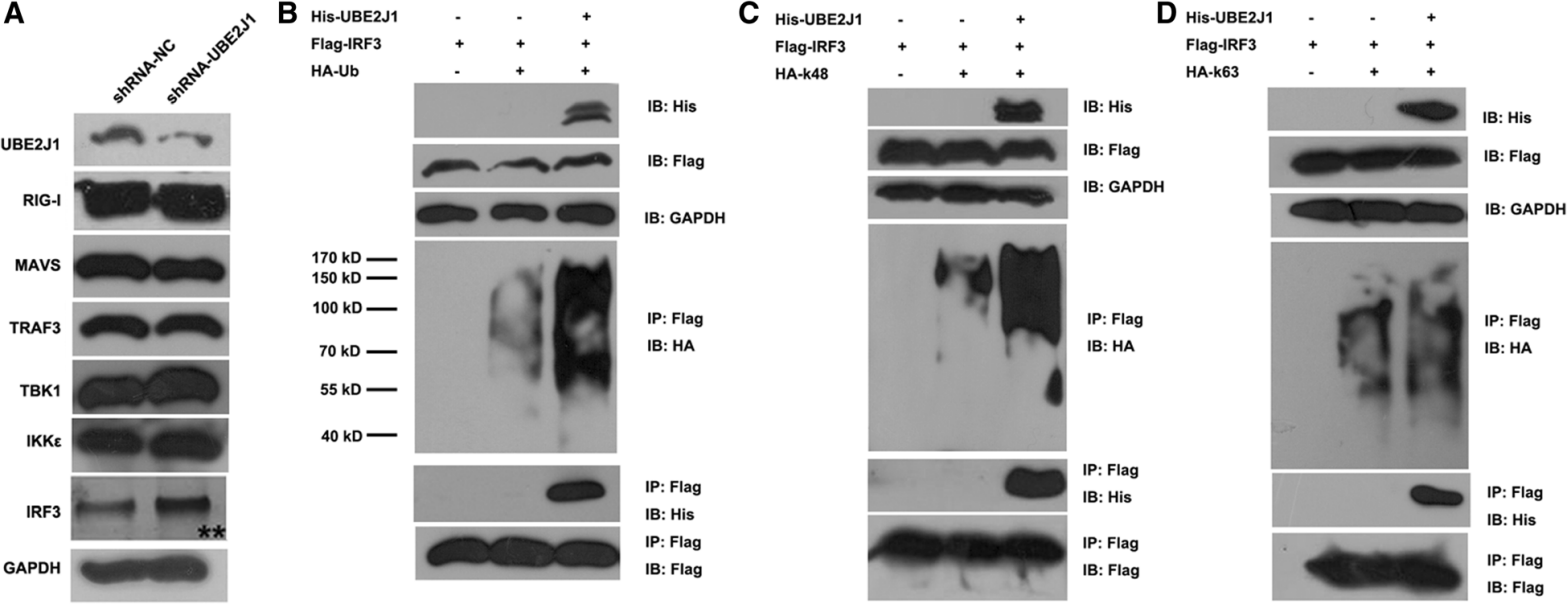
UBE2J1 mainly promotes K48 -linked ubiquitination of IRF3. **a** HEK293T cells were transfected with plasmids of UBE2J1 shRNA (or N.C.) and then infected with DENV for 24 h. RIG-I, MAVS, TRAF3, TBK1, IKKε, and IRF3 protein levels were detected by immunoprecipitation with indicated primary antibodies. The increased IRF3 protein level was indicated by stars (**). (**b**-**d**) UBE2J1 interacted with IRF3 and facilitated IRF3 ubiquitination. HEK293T cells were transfected with Flag-IRF3, HA-Ub (**b**), (or HA-K48-Ub (**c**)/HA-K63-Ub (**d**)), along with UBE2J1-His or control plasmids for 24 h. Cells were then treated with MG132 for 6 h before harvesting the cells lysates. Distinct types of ubiquitinated IRF3 proteins were detected by immunoprecipitation and western blotting with indicated tag antibodies

To confirm that UBE2J1 targets IRF3 to negatively regulate virus-triggered signaling, the protein levels of IFN pathway members were measured by western blot in DENV infected, UBE2J1 silenced and control HEK293T cells. Immunoblot analysis showed that the protein level of endogenous IRF3, but not RIG-I, MAVS, TRAF3, TBK1 or IKKε, was significantly increased in UBE2J1 silenced cells compared with controls (Fig. 5a). This suggests that UBE2J1 may target IRF3 for degradation.

We next test the role of UBE2J1 in ubiquitination of IRF3. His-tagged UBE2J1 was co-immunoprecipitated with Flag-IRF3 by Flag agarose, suggesting that IRF3 interacts with UBE2J1 (Fig. 5b-d). Moreover, the ubiquitin (HA-Ub) chains that conjugated to immunoprecipitated IRF3, were significantly increased in presence of overexpressed UBE2J1 when compared with controls (Fig. 5b). To investigate the types of ubiquitination chains linked to IRF3, we transfected cells with HA tagged ubiquitin mutants K48 or K63, which has only one lysine in ubiquitin at position 48 or 63, respectively. Immunoprecipitation followed by western blots demonstrated that overexpression of UBE2J1 significantly increased K48-linked ubiquitination of IRF3 (Fig. 5c). The K63-linked ubiquitination was also slight increased but not as dramatic as that of K48 ubiquitination (Fig. 5d). Taken together, these findings suggest that UBE2J1 mainly promotes K48-linked ubiquitination of IRF3 and leads to the degradation of IRF3.

## Discussion

In the last few years, ubiquitin modifications have been reported as important regulators of antiviral innate responses including the interferon signaling pathway. For example, TRIM25 mediated K63-ubiquitination of RIG-I is one of the first events following viral infection [6, 24]. Upon activation, RIG-I recruits the several E3s including TRAF6, TRAF2/5, and TRAF3 through the adaptor protein MAVS. Once TRAF6 and RIP1 are K63 polyubiquitinated, they recruit the TAK1 and IKK complexes. Then TAK1 phosphorylates and activates the IKK complex, activating IKKβ and lead to IκBα degradation [10, 25, 26]. K63-linked polyubiquitination of TRAF3 promotes IRF3 activation. Moreover, the activation of NF-κB and IRF3 can be triggered through K27-linked polyubiquitination of NEMO via TRIM23 [27]. On the other hand, K48-specific polyubiquitination trigger proteasomal degradation of signaling molecules in RIG-I pathway [28, 29]. For example, E3 RNF125 induces degradation of RIG-I, MDA5, and MAVS [30, 31]. E3 enzymes HOIL-1 L and HOIP mediate linear ubiquitination, in which the C-terminal carboxyl groups of ubiquitin is conjugated to the α-amino group of N-terminus of another ubiquitin, inhibit RIG-I signaling [32, 33].

Since E2 protein families are critical for determination the types and length of ubiquitin chains, we screened the function of 34 human E2 members on DENV infection. We have found that silencing UBE2J1, UBE2B and CDC34 significantly impaired DENV infection. While, silencing UBE2S, UBE2U, UBE2I and UBE2V1 significantly enhanced DENV infection (Fig. 1a). Among them, CDC34 and UBE2J1 are mainly responsible for K48 linked ubiquitination [15]. UBE2S mediates K11 linked ubiquitination [34, 35]. UBE2V1, formed a heterodimer with UBE2N, mediates K63 ubiquitination which are reported to be important in activation of RIG-I and NF-κB signaling [36]. UBE2I, also named as UBC9, are responsible for protein sumolytion [37]. Chiu M. et al. reported that UBC9 interacts with DENV-2 E protein; overexpression of UBC9 could reduce DENV infection in mammalian cells [38]. This is consistent with our result that silencing UBE2I causing an increased viral mRNA transcript copies in HEK293T cells (Fig. 1a). While, another study, using yeast two-hybrid assay, identified that UBE2I interacted with several DENV NS proteins; silencing UBE2I caused a significant decrease in the replication of a DENV replicon. [39]. In addition, Su C et al. suggested that silencing UBC9 (UBE2I) resulted in NS5 degradation [40]. Since the inconsistent conclusions are existed, further studies will be required to dissect the roles of UBE2I in DENV mRNA replication and protein modifications.

It is well known that UBE2J1 is involved in Endoplasmic Reticulum-Associated Protein Degradation (ERAD) process to degrade the incorrect folding proteins on ERs [41]. Two recent CRISPR/Cas9 screening studies suggested that ER-associated multi-protein complexes, N-linked glycosylation and ER associated degradation are required for flavivirus infectivity. UBE2J1 was also identified in their screenings as a pro-viral protein required for DENV and West Nile Virus infection [42, 43]. Our current study further suggested that UBE2J1 is responsible for ubiquitination of transcription factor IRF3, and mediates IRF3 degradation. In addition, IRF3 is also reported to be modified by UBC5 mediated K63-linked ubiquitination; but this modification is required for IRF3 activation, rather than degradation [44].

Since UBE2J1 mediated ERAD process may influence the stability of many proteins, we believed that IRF3 could just be one of the targets that modified by UBE2J1 during DENV infection. The modification of interferon pathway by UBE2J1 could be one possible mechanism for its pro-viral role in DENV infection. To further investigate the potential targets of UBE2J1 during DENV infection, an immunoprecipitation and protein Mass spectrum identification study was performed in DENV infected cells. Ubiquitin activating enzyme E1, several ubiquitin ligases E3s and proteins involved in protein translation (EIF4A2 and EIF4A2) or glycosylation (e.g. Triosephosphate isomerase) were identified that directly binding to UBE2J1 (data not shown). Further study will investigate the functions of UBE2J1 on these potential targets during DENV infection. Also, we will further characterize the roles of other UBE2s that identified in our screening and reveal the potential roles of different types of ubiquitination during RNA virus infection.

## Conclusion

In conclusion, we reported that UBE2J1, one of ubiquitin E2 protein, could facilitate RNA virus replication. UBE2J1 mediates the ubiquitination and degradation of transcription factor IRF3, thereby negatively regulates type one IFN expression. This study could provide new evidences for the function of E2s and direct the development of new antiviral drugs against distinct E2 proteins.

## Additional file

Additional files 1 Oligo-primer sequences for qRT-PCR Assay (DOCX 16 kb)

## Abbreviations

DENV: Dengue virus
E2: Ubiquitin-conjugating enzyme
IRF3-5D: The constitutively active IRF3
RIG-I-N: The active caspase recruitment domain (CARD) containing form of RIG-I
