# Supplementary material for: Ubiquitin-conjugating enzyme UBE2J1 negatively modulates interferon pathway and promotes RNA virus infection
Source: Virol J. 2018 Aug 29;15:132. doi: 10.1186/s12985-018-1040-5 (PMC6114777; doi:10.1186/s12985-018-1040-5)
Supplement: Supplementary file 1 — Oligo-primer sequences for qRT-PCR Assay (DOCX 16 kb) [file 12985_2018_1040_MOESM1_ESM.docx]

**Additional File 1**. Oligo-primer sequences for qRT-PCR Assay.

|  |  |
| --- | --- |
| Target | Sequence（5’→3’） |
| DENV E | 5’-CATTCCAAGTGAGAATCTCTTTGTCA-3’ |
|  | 5’-CAGATCTCTGATGAATAACCAACG-3’ |
| Human *β-actin* | 5’-GGGCATGGAGTCCTGTGGCA-3’ |
|  | 5’-GGGTGCCAGGGCAGTGATCTC-3’ |
| *UBE2J1* | 5’-AGCGACCTGCCATGGAGAC-3’ |
|  | 5’-TGGTGGCAGCACTATTCGTC-3’ |
| ZIKV E | 5’-GCTGCCCAACACAAGGTGAA-3’ |
|  | 5’-ATGTCACCAGGCTCCCTTTG-3’ |
| H1N1 HA | 5'-TTCTAACCGAGGTCGAAACG-3' |
|  | 5'-ACAAAGCGTCTACGCTGCAG-3' |
| SeV L | 5'-TGCCCTGGAAGATGAGTTAG-3 |
|  | 5'-GCCTGTTGGTTTGTGGTAAG-3' |
| *IFNβ* | 5’-CATTACCTGAAGGCCAAGGA-3’ |
|  | 5’-CAGCATCTGCTGGTTGAAGA-3’ |
| *ISG15* | 5’-ACGCCTTCCAGCAGCGTCTG-3’ |
|  | 5’-CGCATTTGTCCACCACCAGCA-3’ |
| *MxA* | 5’-AAGAGCCGGCTGTGGATATG-3’ |
|  | 5’-TTTGGACTTGGCGGTTCTGT-3’ |
